# Cryopreserved Aortic Homograft Replacement in Pediatric Patients: A Single-Center Experience with Midterm Follow-Up

**DOI:** 10.3390/children12060661

**Published:** 2025-05-22

**Authors:** Mustafa Kemal Avşar, Yasin Güzel, Barış Kırat, İbrahim Özgür Önsel, Deniz Yorgancılar, İlker Kemal Yücel, Cenap Zeybek, İbrahim Savaş Yıldırım

**Affiliations:** 1 Department of Cardiovascular Surgery, Faculty of Medicine, Çukurova University, 01330 Adana, Türkiye; yasin.guzel@cu.edu.tr; 2 Department of Anesthesiology and Reanimation, Medicana International Istanbul Hospital, 34212 Istanbul, Türkiye; baris.kirat@medicana.com.tr (B.K.); ibrahim.onsel@medicana.com.tr (İ.Ö.Ö.); 3 Department of Thoracic Surgery, Medicana International Istanbul Hospital, 34692 Istanbul, Türkiye; deniz.yorgancilar@medicana.com.tr; 4 Department of Pediatric Cardiology, Cerrahpaşa Faculty of Medicine, Istanbul University, 34098 Istanbul, Türkiye; ilker.yucel@iuc.edu.tr; 5 Department of Pediatric Cardiology, Medipol Mega University Hospital, 34896 Istanbul, Türkiye; cenap.zeybek@medipol.edu.tr; 6 Department of Cardiac Surgery, Medicana International Istanbul Hospital, 34520 Istanbul, Türkiye; isyildirim@medicana.com.tr

**Keywords:** pediatric aortic valve replacement, cryopreserved homograft, structural valve degeneration, aortic root reconstruction, midterm outcomes, reoperation, congenital heart disease

## Abstract

**Objective:** To evaluate early and midterm outcomes of cryopreserved aortic homograft implantation in pediatric patients undergoing aortic valve and root replacement. **Methods:** A retrospective analysis was conducted on 36 pediatric patients aged 2 to 7 years who underwent cryopreserved aortic homograft implantation between January 2016 and December 2024. Indications included complex congenital aortic valve disease, annular hypoplasia, failed Ross procedure, and infective endocarditis. The standard root replacement technique was used under moderate hypothermic cardiopulmonary bypass. Postoperative outcomes were analyzed, including early complications, mortality, echocardiographic parameters, and long-term graft performance. Statistical analyses included the use of chi-square test, the Mann–Whitney U test, and Spearman correlation. **Results:** There was no 30-day mortality. One patient (2.8%) experienced late mortality at year 3, and two patients (5.6%) underwent reoperation at years 4 and 7 due to root aneurysm and severe regurgitation, respectively. Early postoperative echocardiography showed satisfactory hemodynamic performance with a mean gradient of 8.4 ± 3.2 mmHg. At 5-year follow-up, 92.9% of grafts maintained normal function. **Conclusions:** Cryopreserved homografts provide a safe and effective option for pediatric aortic valve replacement in the early and midterm period. However, potential late complications such as structural degeneration or root dilation necessitate long-term surveillance. Advances in decellularized grafts may improve future durability and integration.

## 1. Introduction

Aortic valve and root diseases in children represent a significant surgical challenge due to anatomical complexity, somatic growth potential, and limitations of traditional valve substitutes. Although mechanical valve replacement remains effective, its disadvantages—such as lifelong anticoagulation, increased risk of thromboembolic events, and valve-related complications—are particularly concerning in the pediatric population [1,2]. In this context, cryopreserved aortic homografts (CH) have emerged as a valuable alternative in selected pediatric patients. Their low thrombogenicity, reduced susceptibility to infection, and favorable hemodynamic properties support their clinical use [3,4]. Homografts are particularly preferred in cases of active infective endocarditis, complex congenital aortic valve disease with annular hypoplasia, or contraindications to anticoagulation therapy [5,6].

Moreover, homografts are widely used in reoperative scenarios following failed Ross procedures, in aortic root reconstructions, and in neonates or small children for whom prosthetic valves are not feasible due to size limitations [7,8]. Despite concerns regarding long-term durability and availability, midterm outcomes in children remain promising, with acceptable rates of structural valve deterioration [9,10]. Given the challenges posed by small annular dimensions and growth potential in this age group, homografts offer a viable solution due to their infection resistance and the absence of anticoagulation requirements. While cryopreserved homografts have been studied in pediatric populations, data on their use in very young children (2–7 years) with specific indications such as failed Ross procedures or active endocarditis remain limited, particularly in the context of regional healthcare settings like Turkey. In this study, we present our single-center experience of aortic homograft implantation in 36 pediatric patients aged 2 to 7 years. Our objective is to evaluate the surgical indications, early and midterm outcomes, and the clinical role of homografts in pediatric aortic valve and root surgery.

## 2. Materials and Methods

**Figure 1:**
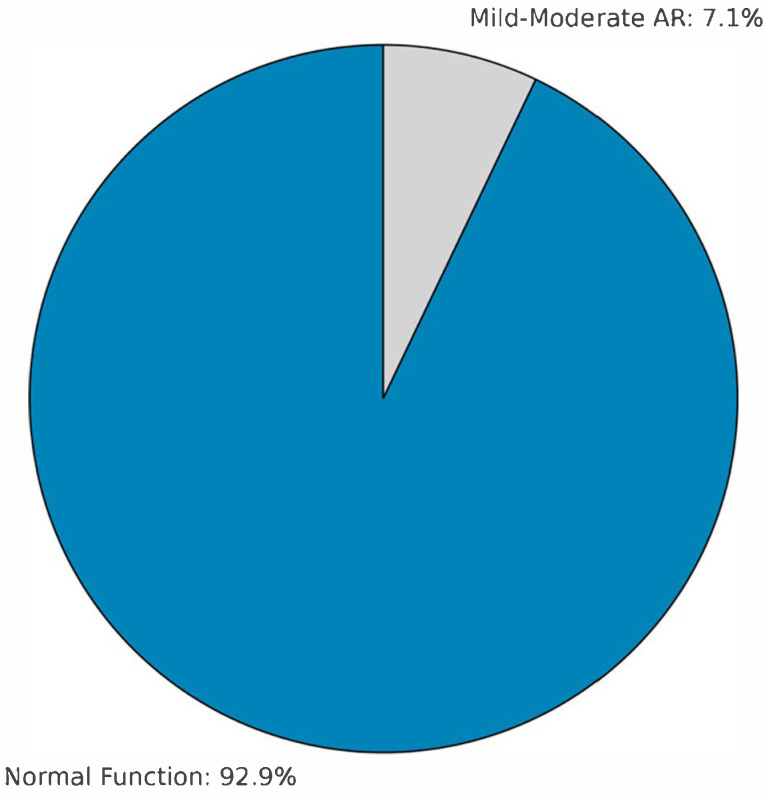
Valve function status at latest follow-up in 36 pediatric patients after aortic homograft implantation. This pie chart represents the valve function status at the latest follow-up in 36 pediatric patients after cryopreserved aortic homograft implantation, showing that 92.9% maintained normal function, and 7.1% exhibited mild-to-moderate aortic regurgitation (AR).

This retrospective study includes 36 pediatric patients aged 2 to 7 years who underwent CH implantation between January 2016 and December 2024 (Figure 1). Patient selection was based on clinical indications warranting homograft use, such as active infective endocarditis, complex congenital aortic valve disease with annular hypoplasia, contraindications to anticoagulation, or failed prior surgical interventions. One case was referred in the 7th postoperative year with severe aortic regurgitation (AR) and underwent homograft re-replacement. The surgical technique involved full root replacement using cryopreserved aortic homografts prepared according to standard protocols. Intraoperative sizing and coronary reimplantation were meticulously performed considering the small anatomical dimensions of the patients. All procedures were carried out by an experienced pediatric cardiovascular surgery team under moderate hypothermic cardiopulmonary bypass.

Early outcomes were assessed by 30-day mortality, postoperative complications (bleeding, arrhythmia, heart block), and echocardiographic valve performance (gradients and regurgitation grades). Mid- and long-term outcomes were evaluated based on structural valve deterioration, reoperation, and survival rates during follow-up (mean 36 months, range 6–84 months). Data were collected from patient charts and a prospectively maintained surgical database. Kaplan–Meier survival analysis was used for statistical evaluation, and a *p*-value of <0.05 was considered statistically significant. The study was approved by the institutional ethics committee (Medicana International Beylikduzu Hospital Protocol No: 2025-045; date: 15 March 2025).

## 3. Results

The demographic characteristics and surgical indications of the 36 patients were analyzed. The mean age was 4.3 ± 1.6 years, with 60% (n = 22) being male. Surgical indications are summarized in Table 1. The average homograft diameter was 13.2 ± 2.1 mm, reflecting the need for anatomical adaptation in this young cohort. The mean cardiopulmonary bypass time was 112 ± 35 min, and the mean aortic cross-clamp time was 91 ± 28 min.

**Figure 2:**
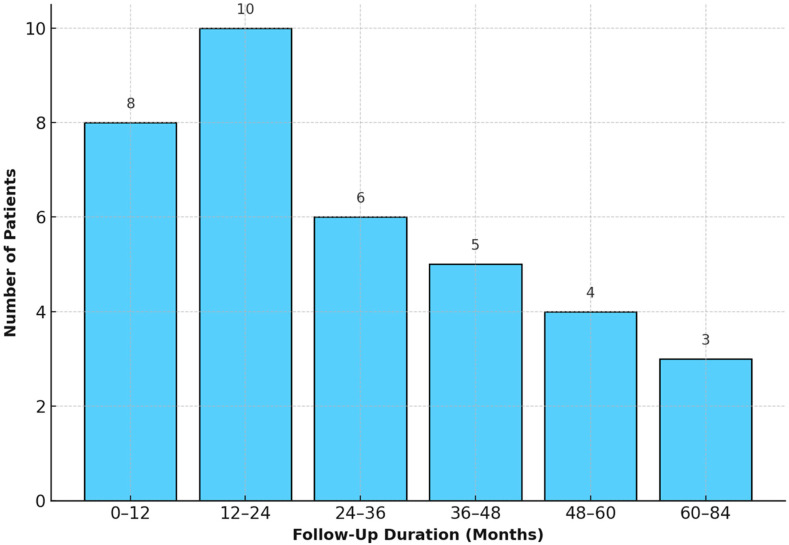
Distribution of follow-up durations among 36 pediatric patients after cryopreserved aortic homograft implantation. This histogram illustrates the distribution of follow-up durations (in months) among 36 pediatric patients who underwent cryopreserved aortic homograft implantation, with patient counts indicated above each bar.

The distribution of follow-up durations among patients is illustrated in Figure 2.

Of the 30 patients with congenital aortic stenosis with annular hypoplasia (n = 25) or valve dysfunction with small annulus (n = 5), 28 underwent root replacement as their initial operation, while 2 had prior balloon valvuloplasty that failed to relieve obstruction.

**Figure 3:**
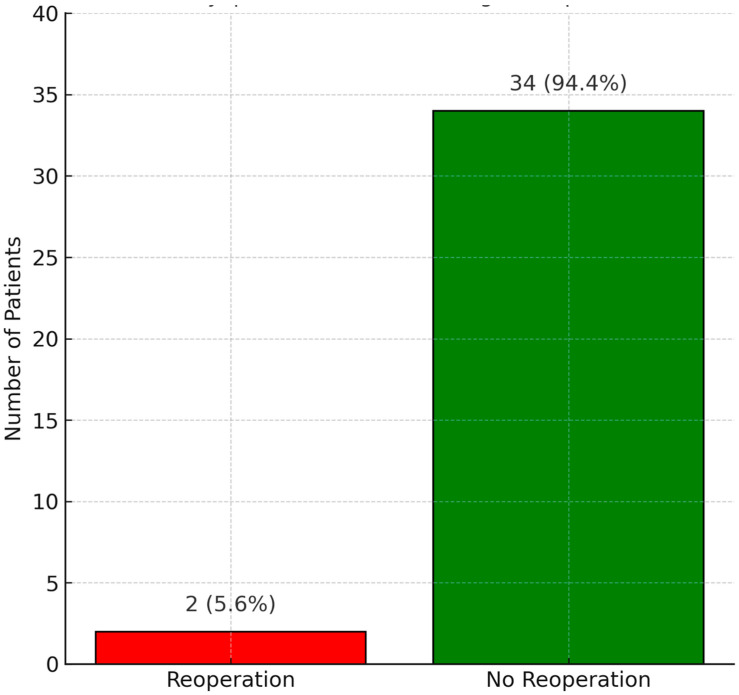
Reoperation status in 36 pediatric patients after cryopreserved aortic homograft implantation. This bar plot summarizes the reoperation status in 36 pediatric patients after cryopreserved aortic homograft implantation, showing that only 2 patients (5.6%) required reoperation during the follow-up period.

**Figure 4:**
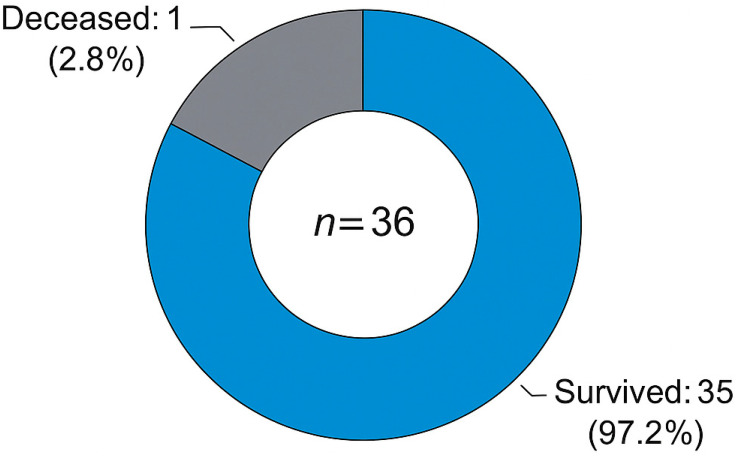
Survival status at follow-up in 36 pediatric patients after cryopreserved aortic homograft implantation. This donut chart illustrates the survival status at follow-up in 36 pediatric patients after cryopreserved aortic homograft implantation, showing that one patient (2.8%) died during late follow-up while 35 patients (97.2%) survived.

**Figure 5:**
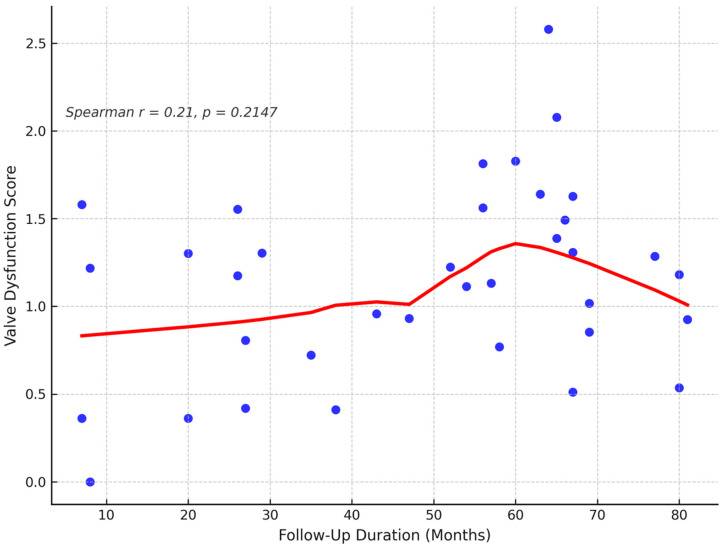
Spearman correlation between follow-up duration and valve dysfunction score in 36 pediatric patients after cryopreserved aortic homograft implantation. This scatter plot illustrates the non-parametric correlation between follow-up duration (months) and valve dysfunction score in 36 pediatric patients. A mild, non-significant positive trend is demonstrated using LOESS regression (red line), with a Spearman correlation coefficient of r = 0.21, *p* = 0.2147, indicating a weak and statistically non-significant association.

The reoperation status during the follow-up period is shown in Figure 3. Only two patients (5.6%) required reoperation: one due to root aneurysm at year four, and one due to severe aortic regurgitation at year seven. Overall survival at follow-up is illustrated in Figure 4. One patient (2.8%) died during late follow-up, while the remaining 35 patients (97.2%) survived. The correlation between follow-up duration and valve dysfunction score is presented in Figure 5. A mild, non-significant positive trend was identified, with a Spearman correlation coefficient of r = 0.21 (*p* = 0.2147), suggesting a weak association between these variables.

Regarding early outcomes, there was no 30-day mortality (0%), indicating a high safety profile of the procedure in this cohort. Postoperative complications were limited to one case of supraventricular tachycardia (SVT) (2.8%, n = 1), which was successfully managed medically. No bleeding complications were reported. Echocardiographic assessment revealed a mean aortic gradient of 8.4 ± 3.2 mmHg with minimal regurgitation (<5% moderate to severe, n = 1), confirming satisfactory hemodynamic performance of the homografts. During the first year, all valves maintained function and structural integrity without the need for reoperation.

At midterm follow-up, 10 patients completed 3-year follow-up without any structural valve deterioration or reoperation. However, one patient died at home during the third year, with the cause of death unknown. This patient was a 4-year-old male with congenital aortic stenosis and annular hypoplasia, with a homograft diameter of 12 mm. The last echocardiographic evaluation at 24 months showed normal valve function (mean gradient: 7.8 mmHg, no regurgitation), and the cause of death remains unknown.

In the long term, one patient underwent reoperation in the fourth year due to homograft root aneurysm, and another—originally operated at an outside center—required homograft replacement in the seventh year due to severe AR. The first reoperation involved a 3-year-old female (homograft diameter: 13 mm; preoperative indication: failed Ross procedure), who developed a root aneurysm at year 4, potentially related to somatic growth. The second reoperation involved a 6-year-old male (homograft diameter: 14 mm; preoperative indication: re-replacement from another center), who developed severe aortic regurgitation at year 7, likely due to structural valve deterioration. At 5-year follow-up (n = 14), 13 patients (92.9%) showed normal valve function with no graft abnormalities; mild–moderate AR was observed in one patient (7.1%). The remaining patients are still under follow-up, with stable valve function and graft integrity at an average of 24 ± 12 months. Kaplan–Meier analysis demonstrated a 5-year survival of 97.2% (35/36) and a 7-year survival of 94.4% (34/36).

## 4. Discussion

Cryopreserved homografts represent a valuable option for aortic valve replacement in pediatric patients due to their low thrombogenic potential and the absence of need for long-term anticoagulation. These valves, derived from human donor tissue, are particularly preferred in cases of active infective endocarditis owing to their biocompatibility and favorable hemodynamic performance [11]. However, their lack of durability compared to mechanical valves and inability to accommodate somatic growth limit their long-term efficacy [11]. In this study, we evaluated early and midterm outcomes in 36 pediatric patients who underwent cryopreserved aortic homograft replacement, accompanied by a comprehensive literature review.

No early mortality was observed in our cohort, while one late mortality case (2.8%) occurred at home three years postoperatively, with an unknown cause. Similarly, Lounsbury et al. reported no early mortality in their series involving 38 pediatric patients undergoing aortic homograft implantation [12]. The low mortality rates in both studies suggest that homograft use in the pediatric population is associated with an acceptable safety profile.

Lounsbury’s study further demonstrated that homograft function remained stable in 74% of patients over a median follow-up of 81 months. A 21% reoperation rate was noted, primarily due to homograft failure at an average of 92 months [12]. In comparison, our study reported only two reoperations (5.6%) during a mean follow-up of 36 months—one due to root aneurysm at year four and another due to severe aortic regurgitation at year seven. By year five, homograft function was preserved in 92.9% of our cases, with no calcification observed. These findings are consistent with the notion that homografts maintain high safety in the early period and that long-term durability is influenced by age, surgical technique, and patient selection. Our study’s low reoperation rate (5.6%) and high 5-year graft function preservation (92.9%) in a younger cohort (2–7 years) suggest that cryopreserved homografts may offer superior midterm outcomes in this specific age group compared to previous studies reporting higher reoperation rates [12,13]. Additionally, the focus on a Turkish cohort provides regional insights into the application of homografts, addressing a gap in context-specific data.

A similar study [14] indicated that younger pediatric patients demonstrated lower calcification tendencies, with reoperation rates remaining under 10% within 5–7 years, which aligns with our results. In a broader series by Binsalamah et al. including 206 pediatric patients, early mortality and early reoperation rates in the homograft group (n = 83) were reported at 4.8% and 3.6%, respectively [13]. Late mortality was 3.6%, while late reoperation occurred in 18.1% of cases, primarily due to graft dysfunction, valve deterioration, or root dilation [13]. These findings support the notion that homografts are safe in the early period but require careful long-term evaluation due to structural durability concerns.

In our cohort, one midterm reoperation and one late at-home death were observed. Most patients followed up to five years showed preserved homograft function, with only one case of root aneurysm requiring reoperation and one patient developing late-onset sudden death. The late mortality case occurred in a patient with normal valve function at the last follow-up, suggesting that the death may not be directly related to homograft failure but highlighting the need to monitor for potential sudden events in this young population. The two reoperations highlight distinct failure modes: the root aneurysm in the 3-year-old patient may be linked to somatic growth exceeding the homograft’s capacity, a known limitation in young children [15], while the severe AR in the 6-year-old patient aligns with structural valve degeneration reported in the literature (e.g., 10–15% at 5 years [13]). Compared to patients with preserved graft function, these cases had smaller homograft diameters (12–14 mm vs. cohort mean of 13.2 mm) and more complex preoperative indications (failed Ross procedure and prior homograft replacement), suggesting that initial sizing and patient selection may influence midterm outcomes. In patients with congenital aortic stenosis and annular hypoplasia or valve dysfunction with small annulus (n = 30), root replacement with cryopreserved homografts was performed as the initial operation in most cases due to the presence of annular hypoplasia and severe valve dysfunction, which precluded effective valve repair. In these cases, the small annular size and complex anatomy made homograft implantation a more feasible option to achieve adequate hemodynamic relief. Echocardiographic evaluation revealed preserved valve function in 92.9% of cases at five years, with only one case of mild-to-moderate regurgitation. These results support the early safety of homografts while highlighting that long-term durability depends on patient selection, graft quality, and surgical technique.

While our study focused on children aged 2–7 years, the applicability of cryopreserved homografts in younger (0–2 years) or older (>7 years) children warrants further discussion. In infants aged 0–2 years, the smaller annular size and rapid growth rate may pose additional challenges for homograft sizing and durability, potentially leading to earlier reoperations. Conversely, children over 7 years may be better candidates for alternative procedures such as the Ross procedure or mechanical valve replacement due to their larger annular dimensions and slower growth rates, which could reduce the need for homografts in these age groups. Further studies with broader age ranges are needed to explore these possibilities.

Recent literature highlights both the advantages and limitations of cryopreserved homografts. Frankel et al. emphasized the low immunogenicity of these grafts but also noted limited long-term durability data and the ongoing risk of early calcification and structural deterioration [3]. The anatomical compatibility and hemodynamic superiority of homografts make them particularly advantageous in young patients where Ross procedures are not feasible. Nevertheless, innovative strategies and individualized follow-up protocols remain necessary to enhance long-term outcomes [16].

Nappi et al. reported that cryopreserved allografts yielded acceptable long-term results in selected patients, with 27% structural valve degeneration and 33.8% reoperation rates over 20 years. All-cause mortality was 32.4%, with valve-related cardiac mortality at 13.8% [14]. These findings suggest limited long-term durability in younger adults. In contrast, our study, with a mean follow-up of 36 months in patients aged 2–7 years, showed only a 5.6% reoperation rate and 2.8% late mortality, indicating strong durability and low complication rates in the early and midterm period.

In a multicenter analysis by Horke et al., previous series linked cryopreserved homografts (CH) to 15–40% early structural degeneration and a high risk of calcification, especially in younger patients whose somatic growth could not be accommodated by the graft, leading to significant late regurgitation or stenosis [15]. Pediatric series using CH also reported 20–40% reoperation rates within 5–10 years, attributed to both degeneration and immune responses. The presence of viable cells in CH may trigger immunologic activation, leading to early graft thickening and degeneration [15]. Fukushima et al. reported a 48.2% reoperation rate in a series of 840 patients over 17 years, mostly due to structural degeneration [10]. In our cohort, only two patients (5.7%) required reoperation by year seven, demonstrating a significantly lower rate and suggesting acceptable midterm durability in selected pediatric patients. One case involved graft dysfunction, the other root dilatation—patterns similar to those in the Fukushima study.

In a randomized controlled trial by El-Hamamsy et al., 10-year survival and freedom from reoperation in Ross patients were 97% and 96%, respectively, compared to 83% and 86% in the homograft group [5]. These results suggest superior durability for the Ross procedure. However, in our study, a cryopreserved homograft was successfully used to treat a failed Ross autograft, demonstrating its continued relevance in such cases. Lupinetti et al. reported a 16% calcification rate and limited annular expansion in homograft recipients, whereas these issues were absent in the autograft group. Reoperation was required in 12% of homograft patients vs. 6.4% in the autograft group [17]. While homografts are safe and effective in the short term, autografts may offer better long-term outcomes in growing children. Therefore, homograft use should be individualized based on age, growth potential, and expected follow-up duration.

Cryopreserved homografts have been clinically used since the 1960s and became widespread in the 1980s with standardized cryopreservation techniques [2,13]. These grafts are stored in liquid nitrogen and offer advantages such as infection resistance and no need for anticoagulation [3]. However, they carry a risk of immune reaction and calcification due to preserved donor cells [18]. Decellularized homografts, developed in the early 2000s, aim to reduce these immune responses [15].

Horke et al. reported a 1.4% 30-day mortality for decellularized homografts—lower than the 3.3–6.9% seen with CH—and suggested that reduced immunogenicity may be responsible [15]. In our series, only 2.8% of patients developed supraventricular tachycardia, with no bleeding or infection. Horke et al. noted no major complications except 0.7% pacemaker implantation in decellularized homografts, possibly due to lower inflammatory responses.

Late complications differ significantly between CH and decellularized grafts. In our cohort, only 7.1% developed mild-to-moderate regurgitation at five years, and two reoperations were required at year seven. Literature suggests 10–15% structural failure for CH at five years [13], exceeding our findings. Dignan et al. proposed that such degeneration is immune-mediated [18], potentially explaining our late regurgitation case. Horke et al. reported a 6% explantation rate for decellularized homografts at five years, primarily due to outgrowth stenosis (62.5%), endocarditis (25%), or root aneurysm (12.5%). These grafts also showed favorable annular adaptation with growth [15], a notable advantage over CH, which lack growth potential and are more prone to late degeneration and calcification [2,13].

Each graft type’s early and late complications stem from distinct processing differences. CH provides rapid access in infected cases [6], while decellularized grafts offer superior immunologic stability [15]. Despite the early and midterm efficacy of cryopreserved homografts, their role may be considered palliative in nature due to the lack of growth potential and the inevitable risk of graft dysfunction, necessitating long-term monitoring and potential reoperation. Future studies should focus on mitigating immune responses and enhancing long-term outcomes through advanced biotechnology and broader pediatric cohorts.

In conclusion, cryopreserved homografts remain a reliable option for aortic valve replacement in pediatric patients, offering low early complication rates. Despite a higher long-term risk of degeneration and calcification compared to decellularized homografts, CH remains a valuable solution in children aged 2–7 years due to their infection resistance and anticoagulation-free profile. We believe that addressing immunogenicity through emerging techniques will further improve homograft outcomes. Sarikouch et al. also reported promising early recellularization data for decellularized valves, suggesting their potential to promote physiological integration and reduce immune response [19].

## 5. Conclusions

In conclusion, this study demonstrates that CH’s are a reliable option for aortic valve replacement in pediatric patients in the early and midterm period. Nevertheless, structural valve deterioration, root aneurysm formation, calcification, and valve degeneration remain concerns in the long term and must be carefully monitored. The potential need for reoperation should always be kept in mind. While recent advances in the literature suggest that decellularized homografts and novel materials supporting somatic growth may help overcome these limitations, cryopreserved homografts continue to serve as a valuable treatment option in the pediatric age group due to their current advantages, such as infection resistance and the lack of anticoagulation requirement. Our findings, focused on children aged 2–7 years, suggest that CH remains a valuable solution in this age group, where rapid growth and small annular dimensions pose unique challenges; however, applicability in younger (0–2 years) or older (>7 years) children requires further investigation due to differing growth rates and alternative surgical options.

Our findings of one late mortality and two reoperations due to root aneurysm and severe regurgitation underscore the need for vigilant long-term surveillance, particularly in very young patients (2–7 years), to detect and manage potential complications such as structural degeneration or growth-related issues early. However, the relatively short follow-up period (mean 36 months) limits our ability to assess long-term outcomes, and extended follow-up studies are needed to confirm the durability of cryopreserved homografts in this population. Based on the findings of our study, we believe that reducing immunogenicity could enhance long-term success in the pediatric population.

## Figures and Tables

**Table 1:** Distribution of surgical indications for cryopreserved aortic homograft implantation in 36 pediatric patients.

Indication	*n*	%
Congenital Aortic Stenosis with Annular Hypoplasia	25	69.44
Bicuspid aortic valve with stenosis	10	27.78
Severe aortic stenosis (unicuspid or others)	8	22.22
Isolated annular hypoplasia	7	19.44
Valve dysfunction (AR) with small annulus	5	13.89
Reoperation after failed Ross procedure	3	8.33
Active infective endocarditis	2	5.56
Re-replacement of homograft due to severe aortic regurgitation (AR)	1	2.78
Total	36	100.00
	36	100.00

## Data Availability

Statistical analyses were performed using SPSS v25.0. Categorical variables were compared using the Chi-square test. Continuous variables were assessed using the Mann–Whitney U test. Correlations between homograft function and follow-up duration were analyzed using Spearman correlation. Kaplan–Meier curves were used for survival and freedom from reoperation estimates. A *p*-value of <0.05 was considered statistically significant.
